# Concurrent validity of countermovement and squat jump height assessed with a contact mat and force platform in professional soccer players

**DOI:** 10.3389/fspor.2024.1437230

**Published:** 2024-07-09

**Authors:** Ludwig Ruf, Stefan Altmann, Katharina Müller, Anja Rehborn, Fabian Schindler, Alexander Woll, Sascha Härtel

**Affiliations:** ^1^ TSG ResearchLab gGmbH, Zuzenhausen, Germany; ^2^ Institute of Sports and Sports Science, Karlsruhe Institute of Technology (KIT), Karlsruhe, Germany; ^3^ TSG 1899 Hoffenheim, Zuzenhausen, Germany

**Keywords:** adults, vertical jumping, football, testing, accuracy

## Abstract

**Purpose:**

The aim of this study was to assess the concurrent validity of a contact mat against force plates to measure jump height in countermovement jump (CMJ) and squat jump (SJ) in professional soccer players.

**Methods:**

23 male professional soccer players performed the CMJ and SJ, which were concurrently recorded using a portable contact mat (SmartJump) and a portable dual force plate system (ForceDecks). Equivalence testing between both systems (contact mat vs. force plate) and the two methods (impulse-momentum vs. flight-time and flight-time vs. flight-time) was performed compared to equivalence bounds of ±1.1 cm for the CMJ and ±1.6 cm for the SJ. Additionally, 95% Limits of Agreement (LoA) and intraclass correlation coefficients (ICC) were computed.

**Results:**

Mean differences for the impulse-momentum vs. flight-time comparison for CMJ [3.2 cm, 95% CI (2.3–4.1)] and SJ [2.7 cm, (1.8–3.6)] were non-equivalent between both systems. LoA were larger than the equivalence bunds for CMJ and SJ, while ICCs were good [CMJ, 0.89, (0.76–0.95)] and excellent [SJ, 0.91, (0.79–0.96)]. As for the flight-time vs. flight-time comparison, mean differences were non-equivalent for the CMJ [1.0 cm (0.8 to 1.2 cm)] and equivalent for the SJ [0.9 cm (0.7–1.1 cm)]. LoA were narrower than the equivalence bounds for CMJ and SJ, while ICCs were excellent [CMJ, 0.995, 95% CI (0.989–0.998); SJ, 0.997, 95% CI (0.993–0.997)].

**Conclusion:**

Our findings indicate that the SmartJump contact mat cannot be used interchangeably with the ForceDecks force platform to measure jump height for the CMJ and SJ.

## Introduction

Vertical jump performance is considered a valid measure of lower limb muscular power which is relevant for the overall performance in a range of team and individual sports (1). Vertical jump performance assessment is commonly used to assess acute responses to load, measure chronic neuromuscular adaptations to load and monitor the return to sport process after injury (2). From a practical perspective, vertical jump performance assessments are generally easy to administer with large squads, time efficient, and generally non-fatiguing, which are important considerations when implementing neuromuscular testing within high-performance environments (3). Thus, the assessment of vertical jump performance is now widely adopted within the training process by strength and conditioning coaches and sport scientists.

Two of the most commonly used vertical jump performance tests in both research and practice are the countermovement jump (CMJ) and squat jump (SJ) (1). Both tests are usually used to evaluate the capability to rapidly develop force during dynamic movements. While the CMJ mainly provides information about the capability to quickly produce force in the slow stretch-shortening cycle, the SJ mainly assesses the capability to quickly produce force during a concentric contraction mode (4). Vertical jump performance is most often expressed by reporting jump height as a key performance-related outcome metric for both the CMJ and SJ (3).

To measure jump height for CMJ and SJ, a variety of systems with different calculation methods exist, such as force platforms, contact mats, optoelectronic devices, video cameras, accelerometers, and linear position transducers (5). While force platforms are considered the most accurate method to measure jump height via the impulse-momentum method, and despite increasingly available valid portable force platforms, accessibility for practitioners might still be limited due to associated costs. Thus, practitioners often use alternative field-based systems to assess vertical jump performance. One commonly used field-based method used by practitioners are contact mats that measure jump height via the flight-time method (6). Both devices offer multiple advantages as large groups can easily be tested and they provide immediate feedback avoiding time-consuming post-collection analysis. However, given the different methods to measure jump height and the somewhat conflicting evidence of previous research, it is crucial to assess the concurrent validity of force platforms and contact mats allowing practitioners and researchers to make better informed conclusions regarding the interchangeability of test results (7).

Therefore, the aim of the current study was to assess the concurrent validity of the SmartJump contact mat compared to the ForceDecks force platform in male professional soccer players. Specifically, the research question was: What is the concurrent agreement between the SmartJump contact mat and ForceDecks force platform to measure jump height in the CMJ and SJ. Based on theoretical and empirical biomechanical investigations (6, 8) we hypothesized that jump height will be greater for the contact mat compared to the force plate for both the CMJ and SJ, despite large correlation between both systems.

## Methods

### Experimental approach to the problem

In the present cross-sectional study, professional male soccer players completed a CMJ and SJ to determine jump height. All trials were concurrently recorded via a portable contact mat (SmartJump, Vald Performance) that was placed on top of a portable dual force plate system (ForceDecks, Vald Performance). The force plates were surrounded by a foam rig. A square was marked on the contact mat with the force plates underneath this square to provide an area in which the players should perform the jump. The trial with the highest jump height as measured by the force plate was used for further analyses. All players were familiar with the testing procedures as it is routinely conducted within the training sessions and physical performance assessment. Data were collected in the morning until noon at the start of the pre-season inside the gym.

### Subjects

In total, 23 male soccer players of a professional German soccer club (age 25.9 ± 5.3 years, standing height 182.0 ± 7.7 cm, body mass 76.1 ± 7.9 kg; four to six training sessions and one official match per week) participated in this study. Players were classified as Tier 3 athletes, i.e., highly trained, according to the Participant Classification Framework (9). Players were free from injuries at the time of testing. Data were collected as part of the routine player monitoring procedures so that ethical approval was not required (10). The study conforms nevertheless to the recommendations of the Declaration of Helsinki.

### Procedures

Players performed an individual 5 min warm-up consisting of dynamic mobility exercises and sub-maximal jumps supervised by a practitioner followed by three trials of the CMJ and SJ. All players performed the CMJ first and then the SJ. A 30 s rest between the trials was provided to ensure sufficient recovery. Two different devices (i.e., force plate and contact mat) were used to measure jump height concurrently.

#### CMJ and SJ assessment

For both jumps, players were required to keep their hands held on their hips and were instructed to jump as high as possible. All jumps were performed with players wearing their regular gym shoes. CMJ stance width and countermovement depth were self-selected by the players resulting in a knee flexion of approximately 60°–90°. Players were instructed to land with the lower limbs as extended as possible at an angle of about 180° and on their toes (1). Intra-day reliability statistics for the CMJ across the three trials were as follows: standard error of measurement (SEM) = 1.1, 95% confidence interval (0.9–1.5 cm); intraclass correlation coefficient (ICC; 3,1, two-way random effects, consistency, single measures) = 0.91, 95% confidence interval (0.85–0.95). Intra-day reliability statistics for the SJ cross the three trials were as follows: standard error of measurement (SEM = 1.6, 95% confidence interval (1.3–2.1 cm); intraclass correlation coefficient (ICC; 3,1, two-way random effects, consistency, single measures) = 0.88, 95% confidence interval (0.80–0.93).

#### Force plate system

A portable dual force platform system sampling at 1,000 Hz (FD4000, Vald Performance, Brisbane, QLD, Australia) was used to measure the vertical component of the ground reaction forces. Jump height was derived from the proprietary software (Jump Application v2.0.8245, Vald Performance, Brisbane, QLD, Australia) using the impulse-momentum method which followed the methods as described previously (8, 11). Briefly, centre of mass velocity is calculated by dividing successive samples of impulse by body mass and the velocity at take-off is used to calculate jump height (12). Landmarks of every jump were visually inspected and removed if the start of the movement was incorrectly identified.

#### Contact mat

A portable jump mat sampling at 1,000 Hz (SmartJump, Vald Performance, Brisbane, QLD, Australia) was used to measure the flight time. Jump height was then calculated using the flight time method as described previously (4). Sensitivity of the contact mat describing the deviation of the default threshold that triggers the take-off and landing was set at 95%, as this was determined an optimal value based on pilot testing prior to data collection.

### Statistical analyses

Descriptive statistics are presented as mean ± standard deviation (SD) or 95% confidence intervals (95% CI). Equivalence testing was performed (TOSTER package, version 0.34) to assess the statistical equivalence of jump height between the contact mat and force plate against an absolute difference value of ±1.1 cm for the CMJ and ±1.6 cm for the SJ. These values described the standard error of measurement for jump height across all trials of the force plate for CMJ and SJ and were therefore used to specify the upper and lower equivalence bounds (13, 14). To assess the corelation between jump heights across both systems intraclass correlation coefficients (ICC; 3,1, two-way random effects, consistency, single measures) (15) and 95% Limits of Agreement (16) were computed. The ICC was interpreted using the following thresholds: ≤ 0.50 as poor, > 0.50–0.75 as moderate, > 0.75–0.90 as good, and >0.90–1.00 as excellent. For all statistical tests the significance level was set to 0.05. Analyses were performed with Rstudio (version 1.3.1056, R Foundation for Statistical Computing, Vienna, Austria).

## Results

**Figure 1:**
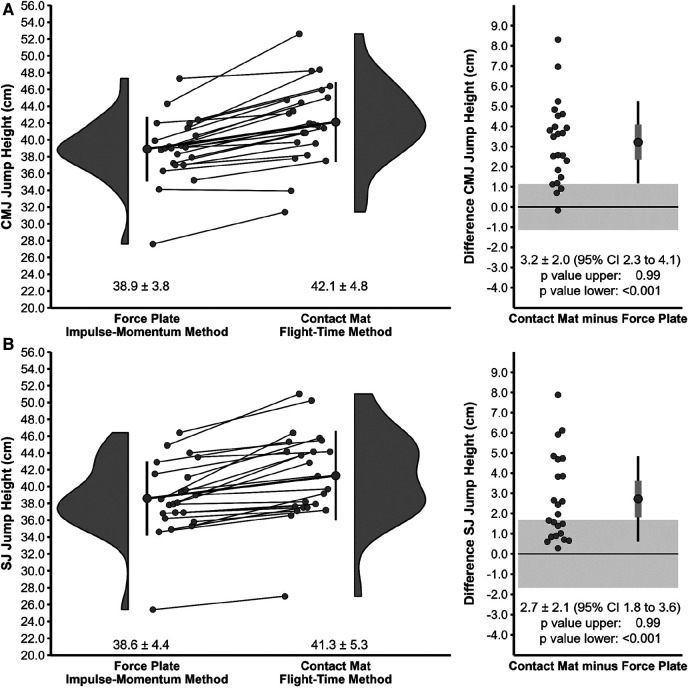
Descriptive statistics for jump height between the force plate system (impulse-momentum method) and contact mat (flight-time method) for the Countermovement Jump (**A**) and Squat Jump (**B**). The left diagram illustrates the absolute mean and SD as well as individual values. The right diagram shows mean, SD (black error bar), and 95% confidence intervals (CI) (grey error bar) as well as individual values for the difference between contact mat and force plate system. The shaded area represents the upper and lower equivalence bounds (see methods).

**Figure 2:**
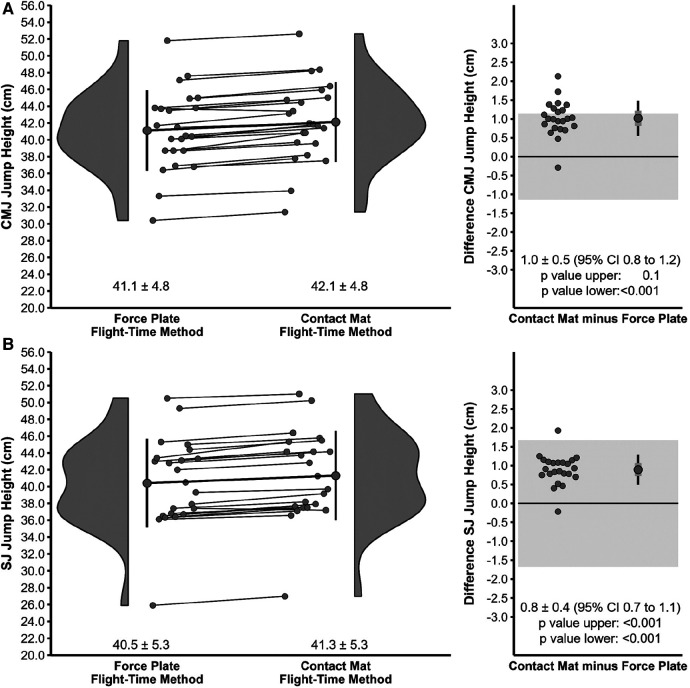
Descriptive statistics for jump height between the force plate system (flight-time method) and contact mat (flight-time method) for the countermovement jump (**A**) and squat jump (**B**). The left diagram illustrates the absolute mean and SD as well as individual values. The right diagram shows mean, SD (black error bar), and 95% confidence intervals (CI) (grey error bar) as well as individual values for the difference between contact mat and force plate system. The shaded area represents the upper and lower equivalence bounds (see methods).

Descriptive statistics for jump height between the force plate system (impulse-momentum method) and contact mat (flight-time method) for the entire sample as well as individual values and differences for each player are illustrated in Figure 1. Figure 2 illustrates the descriptive statistics for jump height between the force plate system (flight-time method) and contact mat (flight-time method) for the entire sample as well as individual values and differences for each player.

Equivalence tests revealed non-significant comparisons between the force plate system (impulse-momentum method) and contact mat (flight-time method) for both CMJ and SJ (see Figure 1). As for the comparison between the force plate system (flight-time method) and contact mat (flight-time method), there was a non-significant difference for the CMJ, but a significant difference for the SJ rejecting the presence of effects more extreme than the pre-defined equivalence bounds of ±1.6 cm (see Figure 2).

Intraclass correlation coefficients ranged from good for the CMJ [ICC = 0.89, 95% CI (0.76–0.95)] to excellent for the SJ [0.91, 95% CI (0.79–0.96)] for the comparison between the force plate system (impulse-momentum method) and contact mat (flight-time method). Excellent intraclass correlation coefficients were evident for the CMJ [0.995, 95% CI (0.989–0.998)] and SJ [0.997, 95% CI (0.993 to 0.997)] for the comparison between the force plate system (flight-time method) and contact mat (flight-time method).

**Figure 3:**
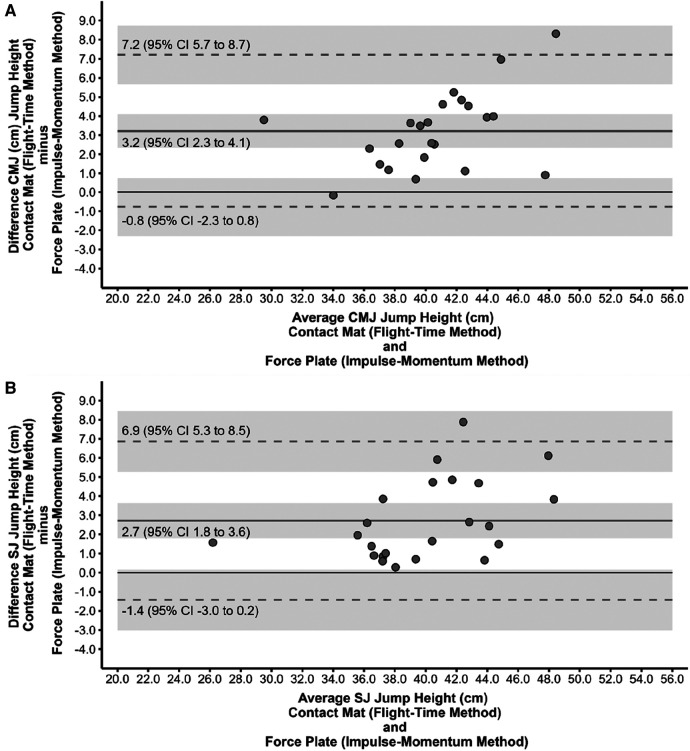
Scatter plot of the limits of agreement between the force plate (impulse-momentum method) and contact mat (flight-time method) system for the countermovement jump (**A**) and squat jump (**B**). Mean difference is represented by the grey horizontal line, whereas the grey dashed lines are the 95% Limits of Agreement. The solid black line indicates zero mean difference. Mean difference and Limits of Agreement are surrounded by 95% confidence intervals (CI) as indicated by the shaded area.

**Figure 4:**
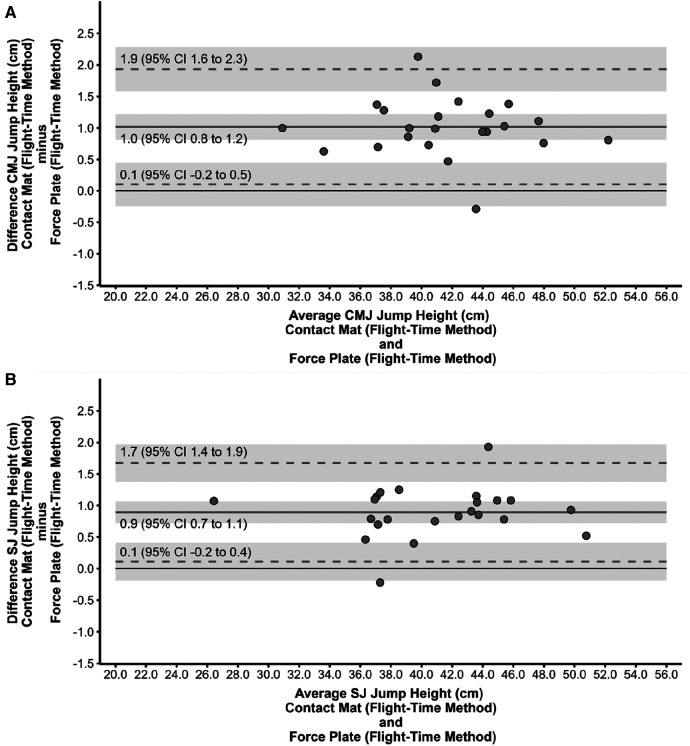
Scatter plot of the limits of agreement between the force plate (flight-time method) and contact mat (flight-time method) system for the countermovement jump (**A**) and squat jump (**B**). Mean difference is represented by the grey horizontal line, whereas the grey dashed lines are the 95% Limits of Agreement. The solid black line indicates zero mean difference. Mean difference and Limits of Agreement are surrounded by 95% confidence intervals (CI) as indicated by the shaded area.

Figures 3, 4 show the scatter plots of the 95% Limits of Agreement, which represent the range within which most differences between the force plate (impulse-momentum and flight-time method, respectively) and contact mat (flight-time method) system measurement fall, for CMJ (panel A) and SJ (panel B).

## Discussion

The purpose of this study was to investigate the concurrent validity of the SmartJump contact mat compared to the ForceDecks force plates to measure jump height in the CMJ and SJ in professional adult soccer players. Our findings revealed that jump height was significantly lower for the force plate system when using the impulse-momentum method compared to the contact mat, although intraclass correlation coefficients between both systems was good to excellent. Similarly, when using the flight-time method for both systems, jump height was slightly higher for the contact mat. Our data indicate that the contact mat cannot be used interchangeably with a force platform to measure jump height for the CMJ and SJ.

Most studies within the scientific literature reported that jump height is systematically higher, on average, for CMJ and SJ when measured with contact mats compared to force platforms (17–20), with few exceptions (21). The magnitudes of the differences in jump height between contact mats and force platforms greatly depend on the specific contact mat with differences of up to 16 cm (20). In the only study using the same contact mat in our study, Reeve et al. (19) observed average jump height differences of 4.5 cm for the CMJ and 1.8 cm for the SJ (compared to our values of 3.2 cm and 2.7 cm, respectively), compared to a Kistler force platform. Additionally, there was a large between-subject variability in the differences between the contact mat and force platform as indicated by the relatively large Limits of Agreement for both CMJ (−0.8 to 7.2 cm) and SJ (−1.4 to 6.9 cm) reaffirming previous research (17). This further highlights the limited construct validity of contact mats against force platforms on an individual level.

There is one major explanation as to why jump height is, on average, higher when measured with a contact mat compared to the force platform using the impulse-momentum method. The different mathematical methods to calculate the jump height mostly contribute to the differences between both systems (6). That is, jump height derived from contact mats is based on the flight-time method which takes advantage of the biomechanical principle of uniform acceleration of an athlete when leaving the contact mat (22). Critically, this method assumes that the position of the centre of mass is the same at take-off and landing of the jump (8). Usually, the knee and ankle joints are fully extended during take-off, but slightly bend during landing. This increases flight time artificially and thus the true jump height is overestimated.

Less obvious are the explanations as to why jump height is, on average slightly higher when measured with a contact mat compared to the force platform using the flight-time method as well. Given the differences in hardness and sampling frequency between the contact mat and the force platform they likely identified take-off and landing at different time points (23). Therefore, flight time and in turn jump height might not be identical. Importantly, although we performed pilot testing to determine the optimal sensitivity of the contact mat, we can not fully rule out that take-off and landing were always correctly identified by the contact mat and force platform given our set-up (i.e., contact mat on top of the force platform).

Despite substantial differences in jump height between contact mats and force platforms, previous studies reported almost perfect correlation between both assessment system (18, 20). This is of particular usefulness as jump height values derived from the contact mat might be corrected using a linear equation potentially allowing the interchangeability of jump height values across both assessment systems. However, our data suggested that correlation between the SmartJump contact mat and ForceDecks force platform using the impulse-momentum method was somewhat weaker, ranging from good for the CMJ [ICC = 0.89, 95% CI (0.76–0.95)] to excellent for the SJ [ICC = 0.91, 95% CI (0.79–0.96)]. The lack of a perfect correlation between both assessment systems consequently prevents the computation of a linear regression model with a very narrow residual error term. This further underscores the limited construct validity when comparing jump heights derived from a force platform using the impulse-momentum method with those from a contact mat using the flight-time method. Conversely, correlation between the SmartJump contact mat and ForceDecks force platform using the flight-time method was almost perfect, allowing the application of a correction equation:

CMJ jump height criterion = (1.0024 × Jump Height Contact Mat)—1.1201, and

SJ jump height criterion = (0.9903 × Jump Height Contact Mat)—0.4934.

Lastly, from a practical perspective contact mats provide only information on the performance outcome and are therefore somewhat limited when aiming to assess the underlying neuromuscular performance and acute responses to load. In contrast, force plates are more readily available and accessible than ever before but require more technical understanding and rigorous application to gather high-quality data within the training process.

### Practical applications

Contact mats are often used as a field-based method by practitioners to assess vertical jump performance because they offer several advantages for assessing large groups in a short period of time. However, practitioners need to consider that the contact mat used in this study (i.e., SmartJump) demonstrated poor construct validity compared to the ForceDecks force platform. Specifically, jump height was, on average, systematically higher for the contact mat compared to the force platform using the impulse-momentum method for both CMJ and SJ, although correlation was good to excellent between both systems. Given the almost perfect correlation between the flight-time methods of the SmartJump contact mat and ForceDecks force platform, practitioners might apply the correction equations presented above if the have to change the measurement system regularly. Nevertheless, practitioners are advised to not directly compare data between both systems and to consistently track performance on the same measurement system, particularly for longitudinal studies to allow for comparability of the collected data.

## Data Availability

The datasets presented in this article are not readily available because the datasets for this article are not publicly available because of legal reasons. Requests to access the datasets should be directed to the corresponding author (stefan.altmann@kit.edu). Requests to access the datasets should be directed to Stefan Altmann, stefan.altmann@kit.edu.
